# The Characterization of Modified Starch Branching Enzymes: Toward the Control of Starch Chain-Length Distributions

**DOI:** 10.1371/journal.pone.0125507

**Published:** 2015-04-13

**Authors:** Cheng Li, Alex Chi Wu, Rob Marc Go, Jacob Malouf, Mark S. Turner, Alpeshkumar K. Malde, Alan E. Mark, Robert G. Gilbert

**Affiliations:** 1 Tongji School of Pharmacy, Huazhong University of Science and Technology, Wuhan, Hubei, China; 2 Centre for Nutrition and Food Science, Queensland Alliance for Agriculture and Food Innovation, The University of Queensland, Brisbane, QLD, Australia; 3 School of Agriculture and Food Sciences, The University of Queensland, Brisbane, QLD, Australia; 4 School of Chemistry and Molecular Biosciences, The University of Queensland, Brisbane, QLD, Australia; National Institute for Medical Research, Medical Research Council, London, UNITED KINGDOM

## Abstract

Starch is a complex branched glucose polymer whose branch molecular weight distribution (the chain-length distribution, CLD) influences nutritionally important properties such as digestion rate. Chain-stopping in starch biosynthesis is by starch branching enzyme (SBE). Site-directed mutagenesis was used to modify SBEIIa from *Zea mays* (mSBEIIa) to produce mutants, each differing in a single conserved amino-acid residue. Products at different times from *in vitro* branching were debranched and the time evolution of the CLD measured by size-exclusion chromatography. The results confirm that Tyr352, Glu513, and Ser349 are important for mSBEIIa activity while Arg456 is important for determining the position at which the linear glucan is cut. The mutant mSBEIIa enzymes have different activities and suggest the length of the transferred chain can be varied by mutation. The work shows analysis of the molecular weight distribution can yield information regarding the enzyme branching sites useful for development of plants yielding starch with improved functionality.

## Introduction

Starch is a homopolymer of glucose with a complex hierarchical structure [1]. It has two major components, amylose and amylopectin. Amylose (average molar mass ~ 10^5–6^ Da) has a small number of long branches, while amylopectin (weight-average molar mass ~ 10^7–9^ Da) has a large number of short branches. The glucose units are connected by α-(1→4) glycosidic linkages in the linear glucan chains, from which there are α-(1→6) glycosidic branch linkages.

The functional and nutritional properties of starch are related to its structure [2–8]. For example, starches with higher amylose content or with longer-branched amylopectin have a higher tendency to retrograde, thus slowing down enzymatic degradation in the digestive track [9]. In the case of amylopectin, a higher proportion of short chains, and therefore a larger number of branches, is unfavorable for α-amylolysis. Starches with slow digestion properties reduce the incidence of metabolic diseases, particularly obesity and diabetes, and alleviate associated complications [5, 10, 11]. Furthermore, the portion of starch that resists digestion in the small intestine and reaches the colon (termed resistant starch, RS) is an important substrate for gut fermentation, the products of which include acetate, propionate and butyrate (IUPAC name butanoate). Butyrate has been shown to promote the proliferation of healthy colonocytes and to suppress the development of cancer cells [12].

**Fig 1:**
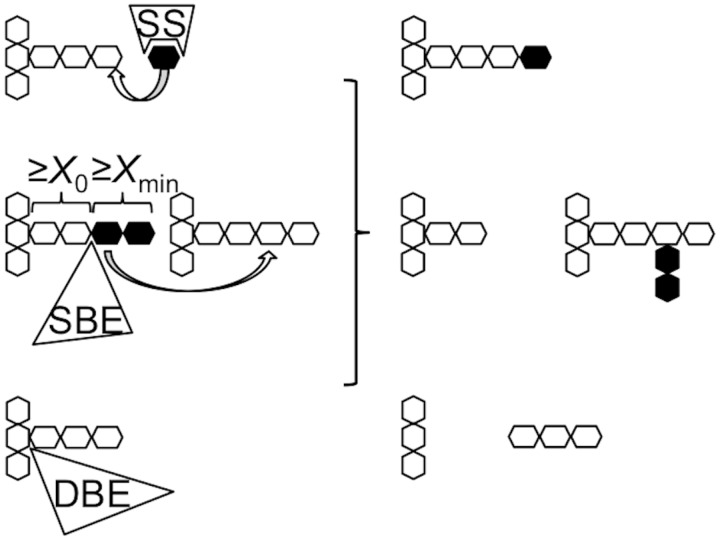
A schematic of starch biosynthesis processes. It shows the key enzymatic steps involved in starch biosynthesis, elongation, branching (for simplicity, only inter-chain branching is shown), and debranching catalyzed by starch synthase (SS), starch-branching enzyme (SBE), and debranching enzyme (DBE), respectively. *X*_0_ and *X*_min_, respectively, are the minimum chain-length constraints of the residual and the transferred segments for the action of SBE.

The biosynthesis of amylose mainly involves granule-bound starch synthase (GBSS) plus some activity of starch-branching enzyme (SBE) for the small number of long chain branches, while for amylopectin, three types of enzyme are essential: soluble starch synthase (SSS), SBE, and debranching enzyme (DBE) (Fig 1). Each type of these biosynthetic enzymes has multiple isoforms [13, 14]. For example, most green plants have two types of SBE, SBEI and SBEII [15, 16]. In addition, in monocots, two classes of SBEII are present: SBEIIa and SBEIIb [17, 18]. Each of these isoforms plays a distinct role in amylopectin biosynthesis [13, 14]. SSS is primarily responsible for the elongation of amylopectin branches, transferring ADP-glucose to the nonreducing end of glucan chains. SBE cleaves an internal α-(1→4) linkage on a donor glucan and transfers the released reducing end to an acceptor chain via an α-(1→6) branch point to form a new branch. For SBE, there are two minimum chain-length constraints on the transferred and the residual segments [19–21]. which have been termed *X*_min_ and *X*_0_, respectively (Fig 1) [22]. These two parameters give the minimum chain length in the chain-length distribution (CLD) of branched glucan produced by SBE. They cannot be differentiated from each other using current characterization techniques because that the minimum chain length could be both the transferred chains and the remaining chains DBE is required for the trimming of improperly positioned branches, which would otherwise delay (or prevent) crystallization of glucans for insoluble starch formation. A number of recent reviews give more details regarding the starch biosynthesis [13, 23–25].

SBE is the only enzyme that generates glucan branches, i.e. it is the only chain-stopping substance for branch growth, and as a consequence SBE has a significant effect on the final structure of the resulting starch. To obtain a desired starch structure, the activity of SBE could be varied by changing its expression level, genetically modifying the activity of the enzyme itself or varying the length of the transferred chain (related to *X*_min_ and *X*_0_, refer to Fig 1 for the definition of *X*_min_ and *X*_0_). Considerable research has focused on changing the expression level of SBE in plants using RNA interference (RNAi). Starches with “high amylose content” (including those containing longer amylopectin chains) have been developed by this method [4, 26–29]. For example, the down-regulation of SBEII results in starches with a higher amylose content and longer amylopectin branches [4, 26, 27]. However, RNAi normally results in the complete removal of all the contribution of one or more SBEs, and the resulting starch has a highly elevated amylose content, which can be disadvantageous, e.g., for mouthfeel and yield. Durum wheat with a modest increase in the amylose content (3%- 22%) was developed by introducing mutations in the *SBEII* gene which alter its activity [30]. This strategy can overcome some of the palatability problems associated with RNAi. Although changing the activity of SBE has some similarities to changing the expression level of SBE, the effect on starch structure is likely to be very different. It is known that the different enzymes form a complex and that the reduction of the expression level of one particular SBE can be complemented by another isoform of SBE *in vivo*. Thus, although a specific enzyme or isoform is removed the plant can still form a functional enzyme complex and work normally. This has been seen in the SBEI lesion plants [31, 32]. However, if the SBE in the complex has reduced activity, a different starch structure would be produced.

Wu *et al*. [22, 33] suggested an alternative method for obtaining starch with longer branches would be to alter the specificities of SBEs, such as the minimal chain-length constraints *X*_0_ and *X*_min_. Using a detailed model to describe the actions of the participating biosynthetic enzymes, it was predicted that a small increase in either quantity has the potential to result in amylopectin in which the number of longer branches was increased significantly. A moderate increase in the amylopectin branch length is not expected to have as significant effect on palatability as when the amylose content is elevated. It is also expected that the starch yield would be similar, as the expression level of SBE could be maintained in the plant. In contrast, the model developed by Wu *et al*. also suggested that eliminating specific isoforms of the essential enzymes would result in reduced starch yield. It is therefore of great interest to determine whether altering the specificities of SBEs while maintaining their expression level in the plant could elevate the nutritional value of starch while maintaining starch yield and palatability. In addition, modified SBEs would provide a powerful tool for understanding both the specificity and biological role of the isoform under study.

Structural information on SBE is limited. The only crystal structure of SBE currently available is that of rice SBEI (PDB codes 3AML, 3VU2 and 3AMK) [34, 35]. This contains features common to members of the α-amylase family of enzymes, such as a central (β/α)_8_ catalytic domain as well as separate C-terminal and N-terminal domains [36]. The catalytic domain is believed to contain a number of subsites, each capable of interacting with one glucose residue of the substrate. In rice SBEI, Tyr235, Asp270, His275, Arg342, Asp344, Glu399 and His467 (rice SBEI numbering) all lie in the central (β/α)_8_ barrel domain and are believed involved in catalysis and substrate binding. Asp344 is believed to serve as a nucleophile in the reaction, whereas Glu399 is responsible for the protonation and deprotonation of the leaving group and attacking oxygen, respectively. This is supported by a range of biochemical data on *E*. *coli* GBE (*EcoGBE*) as well as the crystal structures of apo- and substrate-bound α-amylase and cyclodextrin glycosyltransferase (CGTase) [37–39]. It is also suggested that His275 and His467 are involved in lowering the energy of the transition state in the catalytic reaction, as the corresponding residues do in other α-amylase enzymes [40]. All these key residues lie in the region of subsites –1 and +1. The putative catalytic residues, Asp344 and Glu399, lie in close proximity to a glycosidic linkage between –1 and +1.

In the present paper, we examine what the results of changing a single amino-acid residue in SBE, the objective being to change the CLD by changing activity and/or *X*0 and *X*min, the latter being features of an SBE which is reasonable to suppose might be changed by slight alteration of the binding site. Five conserved amino-acid residues from maize (*Zea mays*) SBEIIa (mSBEIIa) were varied by site-directed mutagenesis and the effects of the mutations on the activity and transferred chain length of mSBEIIa were examined. The five conserved amino-acid residues lie in the binding groove, and have been proposed to play an important role in starch binding and SBE activity. The mutant mSBEIIa enzymes are here expressed in, and purified from, *E*. *coli*. These are then used *in vitro* [19, 41] to form branched glucans from both MAZACA amylopectin and linear α-(1→4)-linked glucans of debranched potato amylose. The CLDs of the branched glucan taken at different times are then analyzed using fluorophore-assisted carbohydrate electrophoresis (FACE) and size-exclusion chromatography (SEC). FACE gives accurate CLD but only when the degree of polymerization (DP) is low (up to ~DP 160) [42]. SEC covers a larger DP range but suffers problems such as band broadening [43]. A combination of these two techniques was therefore used in this study to characterize the *in vitro* branching products. The effects of the selected mutations on the activity and transferred chain length properties of mSBEIIa are discussed and its possible resulting amylopectin CLDs in plants were predicted based the resulting data; while this prediction may or may not accurately reflect what would be found *in planta*, it is of considerable interest, because important functional properties such as digestibility are strongly influenced by the CLD, and a mutant that is predicted to affect this distribution significantly would be a prime target for subsequent *in planta* studies. The combination of the various methods also provides a novel means to obtain information about enzyme branching sites.

## Materials and Methods

### Selection of mutation sites

The sites targeted for mutation were conserved residues in the binding groove of mSBEIIa. To identify residues in the binding groove, the structure of mSBEIIa (NCBI protein ID, AAB67316.1) was modeled using SWISS-MODEL [44, 45]. Rice (*Oryza sativa L*.) SBEI (PDB ID: 3AML) was used as the template. SBEI (PDB ID: 3AML) and mSBEIIa have a sequence identity of 56%. Note that despite the high sequence identity, SWISS-MODEL may not yield an optimal model. For this reason the sequence was also aligned with 14 other branching enzymes (*E*. *coli* GBE, *Mycobacterium tuberculosis* H37RV GBE, rice (*Oryza sativa Japonica Group*) SBEI, wheat (*Triticum aestivum*) SBEI, wheat (*Triticum aestivum*) SBEIIa, wheat (*Triticum aestivum*) SBEIIb, barley (*Hordeum vulgare subsp*. *vulgare*) SBEIIa, barley (*Hordeum vulgare subsp*. *vulgare*) SBEIIb, maize (*Zea mays*) SBEI, maize (*Zea mays*) SBEIIb, pea (*Pisum sativum*) SBEI, pea (*Pisum sativum*) SBEII, potato (*Solanum tuberosum*) SBEII, and Arabidopsis (*Arabidopsis thaliana*) SBEII) by Clustal Omega to help identify conserved sites [46–48].

### Plasmid construction

All of the plasmids were constructed by GeneArt (Germany). The first 20 amino acids were removed from the full-length mSBEIIa sequence during gene construction. This is because after cleavage of the targeting peptide during the import into the plastid, amino acid 21 is known to be the amino terminus of mature mSBEIIa. The mutant and wild-type (WT) mSBEIIa genes were inserted between *XhoI* and *EcoRI* restriction sites in the pRSETA vector (Life Technologies) and the codon usage was also optimized for *E*. *coli* by GeneArt.

### SBE expression

Plasmids were transformed into the *E*. *coli* strain BL21(DE3)pLysS (Novagen), which contains T7 RNA polymerase and T7 lysozyme, for tightly controlled expression. 250 mL cultures were grown at 37°C for 3 h to mid-log phase and induced using 1 mM isopropyl β-D-1-thiogalactopyranoside (IPTG) for 5 h at room temperature. The cell pellets were harvested by centrifugation at 6000 *g* for 10 min and then lysed using BugBuster protein extraction reagent. The *C*-terminal 6×His tag was used to purify SBE from the cell crude extract using the His-Bind kit from Novagen. Amicon ultracentrifugal filter units were then used to concentrate and exchange buffer for the SBE. The SBE was finally stored at -80°C in the buffer containing 0.5 M NaCl, 20 mM Tris-HCl, 5 mM imidazole, and 10% glycerol (final pH 7.9). The final concentration and purity of the enzyme solution were estimated using the bicinchoninic acid (BCA) protein assay kit with bovine serum albumin as the standards and sodium dodecyl sulfate-polyacrylamide gel electrophoresis (SDS-PAGE).

### *In vitro* branching and debranching procedures

To examine the action of the mutant SBEs, the products of *in vitro* branching were then debranched and the resulting CLD examined, using procedures developed previously, with minor modifications [41, 42]. MAZACA amylopectin (from National Starch Pty. Ltd., Lane Cove, NSW, Australia) and debranched potato amylose (average chain length ~ 500, from Fluka) were used as the substrate for the *in vitro* branching reaction. The debranching for potato amylose was performed with the same published procedure [42, 49]. The debranched potato amylose (58 mg) was first dissolved in 5.8 mL of 1 M NaOH at 80°C for 10 min. The pH of the solution was adjusted to 7.4 with 1 M HCl. 3-(*N*-morpholino) propanesulfonic acid (MOPS) buffer (2.9 mL, 500 mM, pH 7.4) was added to the solution and the total volume was then made up to 29 mL with MilliQ water. This solution was then divided into seven aliquots, 4 mL in each. To the remaining 1 mL solution, absolute ethanol (4 mL) was added and mixed gently by inverting the tube, followed by centrifuging at 4000 *g* for 10 min. The supernatant, containing excess salts, was discarded, whereas the desalted precipitate was dissolved in hot water and immediately freeze-dried. The SEC weight distribution obtained from this sample was then processed to give the CLD of the substrate prior to branching with mSBEIIa. Then, 300 μL MilliQ water was added to one of the 4 mL aliquots, which acted as the control. To each of the other 4 mL aliquots, 300 μL of each different mSBEIIa was added. After 3, 6, 9, and 24 h incubation at 30°C, 1 mL samples were taken from each of the aliquots and heated to 98°C for 5 min to stop the reaction. The samples were then desalted and debranched as described above. FACE was used to characterize the *in vitro* branching products from MAZACA amylopectin. The whole procedure of preparing the samples for FACE analysis was similar as the above procedure but the samples were taken after 1, 3, and 6h incubation at 30°C and only 120 μL of mSBEIIa was added to the reaction mixture compared to 300 μL added when preparing SEC samples. These experiments were repeated twice independently.

### Analysis of chain-length distribution of the branched glucan using SEC

The separation of the mixture obtained by debranching the glucan was performed on an Agilent 1100 Series SEC (Agilent Technologies, Waldbronn, Germany) consisting of an isocratic pump, a series of separation columns (GRAM precolumn, GRAM 30, and 1000 analytical columns, Polymer Standard Services, PSS, Mainz, Germany), and a refractive index detector (RID, 235RID-10A, Shimadzu, Kyoto, Japan). The separation columns were held at 80°C in the column oven and the detector was set at 45°C. The eluent used was DMSO containing 0.5% w/w of LiBr. The flow rate was 0.5 mL min^–1^. A series of pullulan standards (Polymer Standard Services, Mainz, Germany) formed by α-(1→4) glycosidic linkages, with varying molecular weights ranging from 342 to 2.35 × 10^6^ was used for calibration, which fully covers the range of molar mass and size of the injected samples. The Mark—Houwink parameters for this eluent at 80°C are *K* = 2.424 × 10^-4^ dL g^–1^ and α = 0.68 [50]. The resulting SEC chromatograms were analyzed using PSS WinGPC Unity software (PSS) and methodology given elsewhere [43]. In short, the RID detector gives the SEC weight distribution, *w*(log *X*): the weight of branches in the interval d(log *X*), *X* being the degree of polymerization (DP). *X* was obtained using universal calibration and the Mark—Houwink equation [51]. While this approach is not especially accurate, we are primarily interested in relative changes; small errors in the absolute values will make no qualitative difference to the overall conclusions. More accurate CLDs are obtained for lower DPs using FACE [52], but this technique cannot be extended to the relatively high DPs in the upper part of the range examined.

### Analysis of chain-length distribution of the branched glucan using FACE

The chain-length distribution of the resulting mixture from MAZACA amylopectin was characterized by FACE after labeling the debranched glucans with the fluorescent probe APTS at their reducing ends, following the method of Wu *et al*. [42], which can characterize up to ~DP 160. FACE gives the number distribution of (debranched) chains, *N*de(*X*), with *w*(log*X*) = *N*2 *N*de(*X*) (the quantitative comparison of experimental FACE and SEC distributions requires corrections for SEC band broadening [51]).

### Enzyme assay

The activities of the mSBEIIa mutants were calculated from the area-normalized FACE results (normalized to the same starch weight used in the branching experiment), giving the rate of incrementing the number of branches. The total number of chains at the beginning and after 1 h incubation with mSBEIIa were calculated based on the method of Nakamura *et al*. [53]. One unit (U) of SBE activity was then defined as the amount (nmol) of new branches produced per min by 1 mg SBE at 30°C. The other conditions for the activities are pH 7.4 and substrate concentration 2 mg mL–1.

### Native PAGE and affinity electrophoresis

For native polyacrylamide gel electrophoresis (PAGE), 5% polyacrylamide (37:1 w/w acrylamide: bis-acrylamide) in 1.5 M Tris-HCl buffer pH 8.8 was used. The debranched potato amylose (2 mg mL–1) was added to the polymerization mixture. mSBEIIa (1.6 μg) was loaded on the gel and electrophoresis was carried out at 100 V constant at room temperature in running buffer (192 mM glycine, 25mM Tris, 1 mM DTT) for 90 min. The migration distances of the protein zones were visualized after staining with Coomassie Brilliant blue.

### Molecular dynamics simulations

All MD simulations were performed using the GROMACS simulation program version 3.3.3 [54], with the GROMOS54A7 force field for protein and starch molecules [55]. Two systems were simulated. The first consisted of a complex of wild-type rice SBEI with maltopentaose, and the second a complex of R342K rice SBEI with maltopentaose. Details of the simulation method are given in SI (S1 Text).

### Estimating starch CLD from a biosynthesis model

The possible effects of changing the activity and *X*_min_ on amylopectin CLD in plants were predicted by the mathematical model of starch biosynthesis developed by Wu *et al*. [22, 33]. Amylopectin CLD with DP up to ~30 can be represented with two enzyme sets: enzyme set (i) and (ii) [33]. The contribution to the amylopectin CLD from each enzyme set is parameterized by *X*_0_, *X*_min_ and β. β is a ratio of the sum of branching enzyme activity divided by that of starch synthase. These enzyme activities are not directly related to specific genetic forms of the enzymes, but rather, to any isoforms that contribute to a range of chains of interest. When applied to a range of chains in the amylopectin CLD, each β describes the ratio of the enzyme activities governing the synthesis of the chain lengths dominating an appropriate range, e.g. β_(i)_ dominates the distribution of the global maximum. There is no differentiation between *X*_0_ and *X*_min_ in this model. Prediction of the amylopectin CLD with modified starch branching enzyme was achieved with a lower β_(i)_. Different values of *X*_min(i)_ and *X*_0(i)_ were also used for calculations.

## Results

### Selection of mutation sites

**Fig 2:**
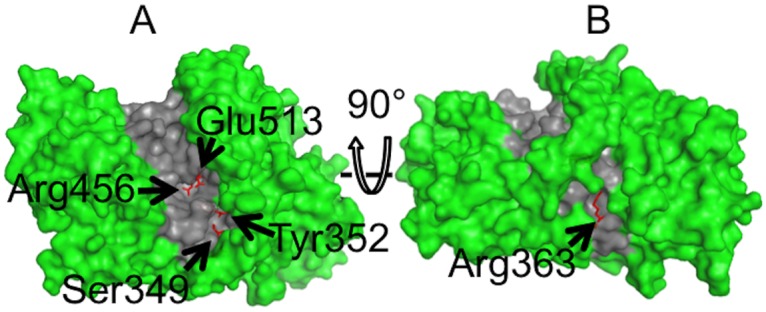
The van der Waals surface of a structural model of wild-type mSBEIIa. The model was generated using SWISS-MODEL with the structure of rice (*Oryza sativa L*.) SBEI (PDB ID: 3AML) as a template. The model, truncated at amino acid 127, is orientated to show the front (A) and rear face (B) of the binding groove. The locations of the mutated residues are indicated by the arrows. The binding groove is highlighted in gray. Figures were generated using pyMOL version 1.6.9 [57].

There are 73 residues completely conserved among the 15 branching enzymes (sequence alignment shown in S1 Fig). After examination of the homology model generated by SWISS-MODEL using the structure of rice (*Oryza sativa L*.) SBEI (PDB ID 3AML) as a template, four conserved residues that lay within the hydrophobic groove (Tyr352, Glu513, Ser349, and Arg456) together with another conserved residue (Arg363) located at the back of the groove were identified as potentially interacting with the glucan and selected for the mutation studies (Fig 2). Arg363 on the back of the groove was mutated, as the glucan substrate may wrap around the enzyme during binding. In this case the mutation of amino acids which lie on the back of the groove may also affect the activity and specificity of mSBEIIa. Four of the selected residues were substituted by similar amino-acid residues, except in the case of Ser349 which was replaced by Phe. The specific mutations were Y352F, E513D, S349F, R363K, and R456K. Conservative mutations were used in order to vary the interactions with the glucan while minimizing the effect on the structure of the enzyme itself. The mutation S349F was introduced in an attempt to create an additional binding site for glucose [56]. Each of the mutants were expressed and purified as described above. The purities were estimated by SDS-PAGE (S2 Fig). The concentrations were determined by the BCA protein assay: WT, 767 μg mL^–1^; Y352F, 950 μg mL^–1^; E513D, 761 μg mL^–1^; S349F, 369 μg mL^–1^; R363K, 531 μg mL^–1^; and R456K, 1083 μg mL^–1^.

### mSBEIIa-catalyzed *in vitro* branching of linear long chains

**Fig 3:**
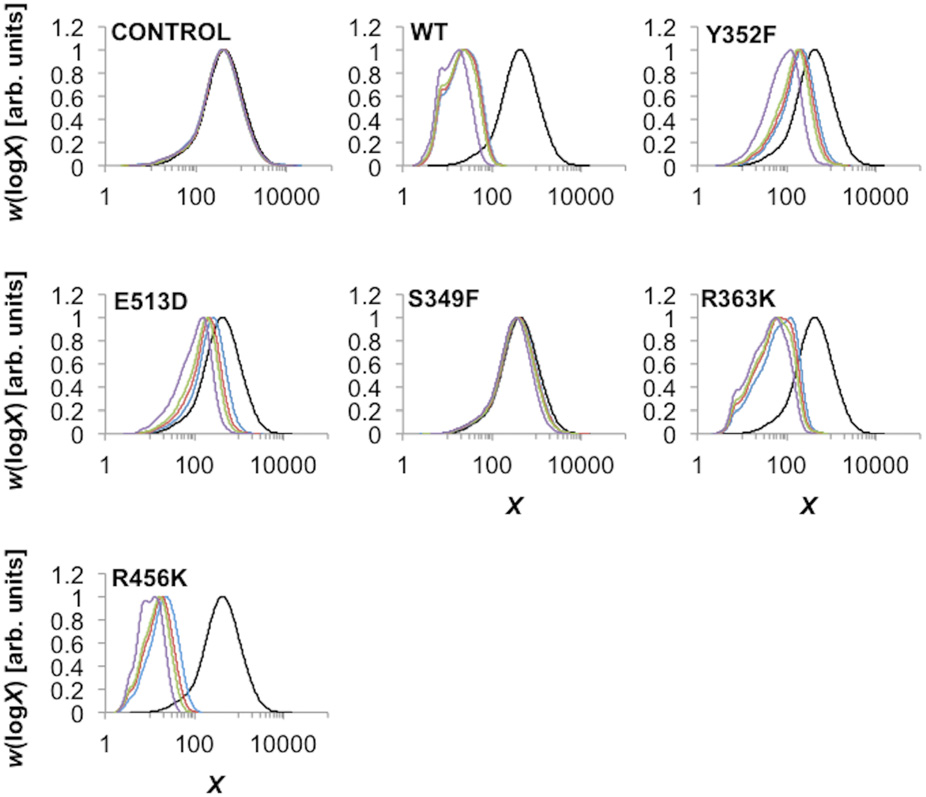
SEC characterization of the branched glucan products. The SEC weight distributions (arbitrarily normalized to the same maximum) are as functions of degree of polymerization, *X*, of the constituent branches of the branched glucan product after incubation with different mSBEIIa for different times. Black, blue, red, green, and purple lines are for CLDs after 0, 3, 6, 9, and 24 h incubation, respectively. The data shown correspond to one of the two independent experiments performed. In each case the difference between the two experiments was small.

SEC weight distributions of the branched glucan derived from the linear debranched potato amylose at various incubation times are given as function of DP *X* in Fig 3. The maxima of the curves were normalized to unity, to give an indication of the relative changes in peak heights.

The SEC weight distribution of the control (no SBE) did not show any shift during the branching procedure. This showed that debranched potato amylose did not undergo spontaneous degradation or retrogradation during the assay procedure.

Significant shifts toward shorter DPs in the WT SEC weight distribution with incubation time were observed. This results from a reduction in the lengths of the glucan chains from the cleavage of new branches. As only the branching enzyme is present, chain elongation is not possible. The shift towards shorter DPs occurred primarily during the first 3 h and became progressively slower after 6, 9, and 24 h. Two peaks were observed, a smaller peak at ~ DP 7 and a larger peak that shifted progressively with time, reaching ~ DP 18 after 24 h incubation.

R363K showed smaller shifts in the SEC weight distribution compared with the WT. Different peaks were observed from the resulting SEC weight distribution. The position of the smallest peak was the same as that in the WT, ~ DP 7. The two other peaks were ~ DP 22 and 58, after 24 h incubation. The largest peak produced by R363K mSBEIIa was observed after 3 h incubation among the 4 time points tested, which was ~ DP 140.

R456K showed somewhat different behavior to the WT. While the peak in the SEC weight distribution at around DP 7 was retained, a peak at ~ DP 3 was also evident. Further, the evolution of the SEC weight distribution towards shorter DP values was faster than that of the WT although this was because a slightly higher concentration of R456K mSBEIIa was used.

Y352F and E513D showed only small shifts in the SEC weight distribution compared with WT. S349F, like the control without enzyme, showed almost no shift in the SEC weight distribution, suggesting the protein was inactive.

### mSBEIIa-catalyzed *in vitro* branching of MAZACA amylopectin

**Fig 4:**
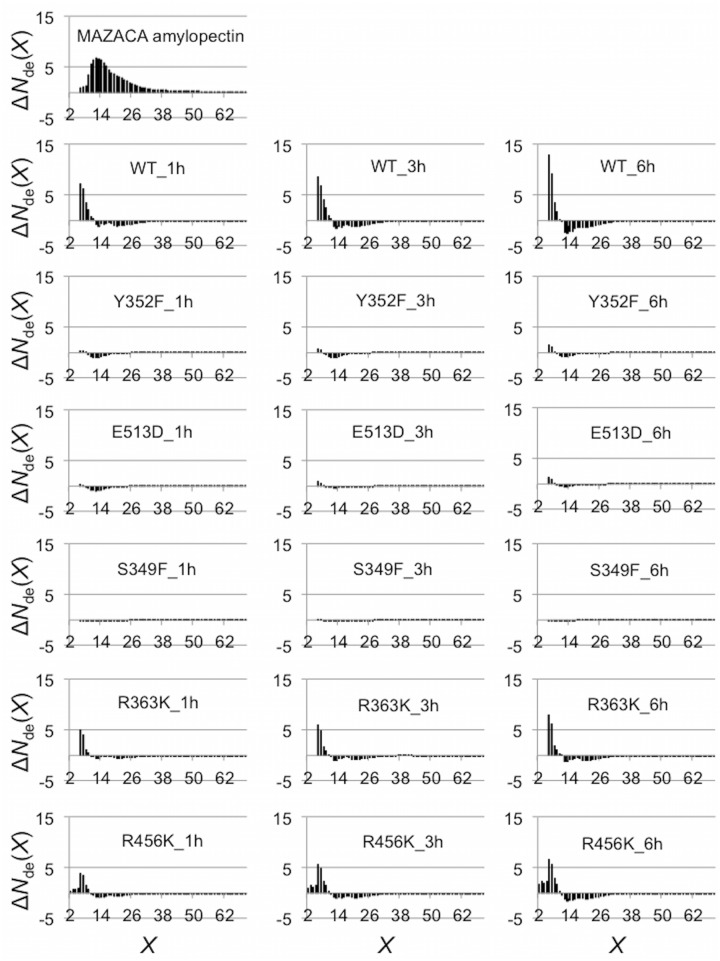
FACE characterization of the branched glucan products. These products were produced by WT mSBEIIa and mSBEIIa mutants using MAZACA amylopectin as the substrate. Top panel: CLD of MAZACA amylopectin expressed as molar % of each chain in MAZACA amylopectin. Lower panels: relative CLD of the products formed by wild-type mSBEIIa and mSBEIIa mutants enzymatic reactions at 30°C for 1, 3 and 6 h were subtracted from that in the substrate MAZACA amylopectin. The total number of chains in the substrate and products were normalized as 100. The data shown here are from one representative experiment chosen from two independent experiments. Both experiments showed the same trend.

The branching characteristics of WT mSBEIIa and mSBEIIa mutants on the branched substrate were investigated using FACE. To analyze the preference of the WT mSBEIIa and mSBEIIa mutants for particular chain lengths during branching, the difference between the debranched chain distribution of the products after exposure to a given form of mSBEIIa to that of the substrate glucan (MAZACA amylopectin) was examined. These difference distributions are presented in Fig 4 on a molar percentage basis (Δ*N*_de_(*X*)) for each of the forms of the enzyme examined. In the case of the WT mSBEIIa, increases in the prevalence of short chain branches were observed up to DP *X* ~ 11. The greatest increase was observed for DP 6, followed by DP 7. The fraction of intermediate and long chains, DP ≥12, decreased. The same pattern of change was observed irrespective of the duration of the enzymatic reaction with mSBEIIa and amylopectin. The fact that no chains having a chain length of DP ≤5 were detected indicates that the minimum chain length that mSBEIIa can produce is 6, suggesting that both *X*_0_ and *X*_min_ are ≥6 (refer to Fig 1 for the definition of *X*_min_ and *X*_0_). The minimum chain length that mSBEIIa can transfer is 12, suggesting that *X*_0_ + *X*_min_ = 12. This means that both *X*_0_ and *X*_min_ = 6.

Three of the mutant forms of mSBEIIa (Y352F, E513D, and S349F) showed much lower activity compared to WT mSBEIIa, with in the case of Y352F and E513D only a slight increase in the proportion of DP 6 and DP 7 chains being observed even after 6 h of incubation. There was nearly no change in the case of S349F. In contrast, the mutants R363K and R456K showed significant activity. R363K showed a similar activity to WT mSBEIIa, with the greatest increase being in DP 6, and there being no increase in chains with DP ≤5. Interestingly, in the case of R456K there was significant increase of chains with DP ≤5 compared to the WT mSBEIIa, although the greatest increase of chains was still with DP 6.

Although we did not investigate the possibility of intermolecular formation of large branched molecules in the present paper, our earlier study [19, 41] in a basically similar system gives some examples of the SEC distributions of the whole (undebranched) molecules, which show conclusively that there is no such effect, as there are no molecules formed which were much larger than the parent ones.

### Activity calculation

The FACE data indicate that Y352F, E513D and S349F have almost no enzymatic activity. The activities of WT, R363K and R456K mSBEIIa were calculated from these data. The average specific activity of R363K and R456K mSBEIIa were 19.0 and 9.5 nmol min–1 mg–1 protein during the first hour incubation, which are 55.5% and 27.8% of that of WT mSBEIIa activity (34.3 nmol min–1 mg–1), respectively.

### Effects of mutation on linear-glucan-binding properties of mSBEIIa

**Fig 5:**
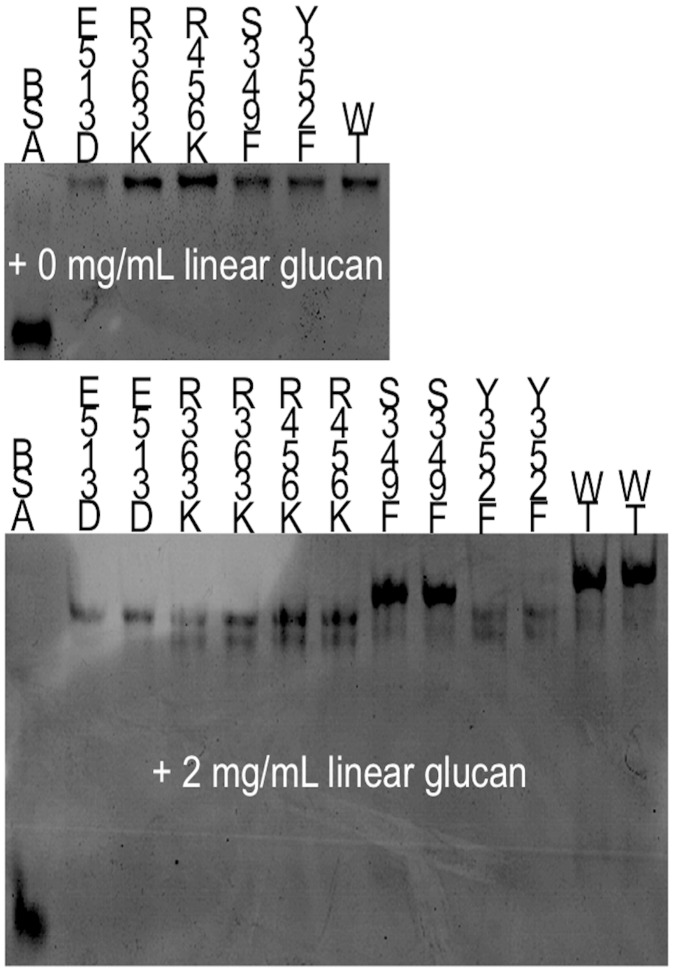
Native affinity gel. It shows the relative migration distance of the WT and mSBEIIa mutants in the presence of 0 mg mL^–1^ and 2mg mL^–1^ linear glucan compared to BSA. BSA was used as the standard for the comparison of migration rate of mSBEIIa. The experiment was repeated twice.

The affinities of the mSBEIIa mutants towards linear branches relative to that of the WT mSBEIIa were assessed using a native affinity gel, the results of which are shown in Fig 5. Debranched potato amylose (2mg mL–1) was added as the substrate. Bovine serum albumin (BSA) was used as a standard in both the gels with and without the linear glucan. The migration rate of BSA is not affected by the presence of the linear glucan. The migration rate and hence the degree of binding was judged from the relative distance between the bands of mSBEIIa and BSA: closer relative distance meant a higher migration rate and lower affinity towards the substrate.

Comparing the gels with and without the linear glucan, it can be seen that WT mSBEIIa and mSBEIIa mutants migrated more slowly in the presence of the linear glucan. In the mutants of mSBEIIa, only S349F mSBEIIa had a similar affinity towards the linear glucan as WT mSBEIIa. All other mutants showed a weaker affinity.

### Molecular Dynamics simulations

**Fig 6:**
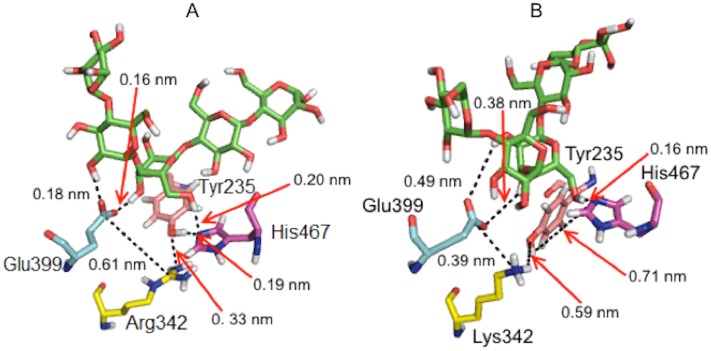
The positions of Glu399, Tyr235, His467 and Arg342 (rice SBEI numbering) with respect to maltopentaose. The positions were produced after 25 ns simulation from (A) the WT rice SBEI and (B) the R342K rice SBEI. The dashed lines indicate distances between key functional groups. The figures were generated using pyMOL version 1.6.9 and orientated such that the backbone of the protein is superimposed [57].

From Fig 4 it is evident that the mutation R456K has a significant effect on the DP of the transferred branches, suggesting a loss of specificity within the active site. To investigate this in more detail, atomistic molecular dynamics (MD) simulations were used to examine possible changes within the active site induced by this mutation. As the structure of mSBEIIa with or without substrate has not been solved experimentally, this element of the work was based on the crystal structure of SBEI from rice (PDB ID 3AML). The residue corresponding to Arg 456 in mSBEIIa is Arg342 in rice SBEI (see S1 Fig for the sequence alignment). The 3AML structure does not contain substrate. For this reason, maltopentaose was docked into the binding groove in the region believed to contain the active site. Fig 6 shows the final positions of the key residues from the wild-type and the R342K mutant with respect to maltopentaose after 25 ns of simulation, together with the distances between key functional groups. The images are aligned such that the backbone of the protein (not shown) is superimposed. In the simulations of WT rice SBEI with maltopentaose the side chains of Glu399 and His467 (Rice SBEI numbering) were found to form stable H-bonds (<2.0 nm) with the maltopentaose. Arg 342 lay in close contact (3.3 nm) with Tyr235, the hydroxyl group of which formed a hydrogen bond with His467, potentially stabilizing the interaction of His 467 with the sugar. Both His467 and Glu399 are known to be important for catalysis (Fig 6A). By contrast, in the mutant form of rice SBEI (R342K), Glu399 and Lys342 moved to within 0.4 nm of each other. Associated with this, Glu399 moved away from the maltopentose and no longer formed stable hydrogen bonds with the sugar (Fig 6B). Tyr235 was also found to have rotated relative to its position in the WT enzyme, and the hydroxyl group of Tyr235 did not form direct contacts with either Lys342 or His467. While His467 did form a hydrogen bond with the sugar, the position of the maltopentose was shifted within the binding pocket. Similar results were obtained in both of the independent runs.

### Starch CLD prediction

**Fig 7:**
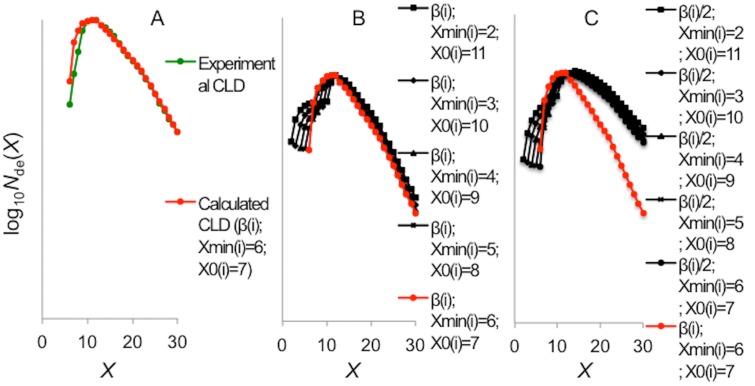
Amylopectin CLD (up to DP 30) prediction. It is predicted from changing the activity and transferred chain length (*X*_0_ and *X*_min_) of SBE (i) based on the Nipponbare endosperm amylopectin CLD. The CLD fitting of the Nipponbare amylopectin experimental CLD (A, green dots) is shown in red dots in panels A, B and C. The parameters used were β_(i)_ = 1.4, *X*_min(i)_ = 6 and *X*_0(i)_ = 7. The black curves were created by changing the *X*_min(i)_ (B) and β_(i)_ together *X*_min(i)_ (C).

The possible effects of changing the activity and *X*_min_ on the final amylopectin CLD in plants were predicted by modifying the parameters of SBE(i) in enzyme set (i) of Nipponbare endosperm amylopectin using the mathematical model of starch biosynthesis developed by Wu *et al*. [22, 33]. The parameters used for fitting enzyme set (i) are *X*_min(i)_ = 6, *X*_0(i)_ = 7 and β_(i)_ = 1.4 (Fig 7A). The predictions for the amylopectin enzyme set (i) fraction of the amylopectin CLD were made by altering the parameters as follows. There are several observations from the observed amylopectin fraction of the starch CLD which can be further understood with these simulations. Variation A: varying the value of *X*_min_ from 2 to 11, but with the sum of *X*_0_ and *X*_min_ equal to 13. The first observation is that the maximum in the starch CLD is stable at DP 13 (Fig 7B). It appears that it is the sum of *X*_min_ and *X*_0_ that determines the position of the maximum; this is also the case for the predictions with the reduced β_(i)_ value (Fig 7C). The second observation is that short chains with DP <13 become even shorter with respect to the selection of *X*_min_ and *X*_0_. It can be seen that as *X*_min_ and *X*_0_ are changed, a bump appeared between the value of *X*_0_ and *X*_min_, which ultimately became a local maximum. It appears that when the difference between *X*_min_ and *X*_0_ is at the greatest, the amount of shorter chains is at the largest. The third observation is that an increase in the relative number of long chains beyond DP 13, which reached a maximum of 110% of the reference, when the difference between *X*_min_ and *X*_0_ is greatest (Fig 7B). Variation B: reducing β_(i)_ to 50% of that of the reference together with varying *X*_min_ from 2 to 11 (Fig 7C). Compared to the reference starch CLD (Fig 7A), this predicted starch CLD has a significantly elevated relative number of chains with larger DPs. This predicted trend is consistent with that reported previously, when rice SBEIIb was down-regulated [27].

## Discussion

### SEC characterization of the *in vitro* branching reaction reveals the importance of N-terminal in determining the transferred chain length of SBE

The SEC weight distributions from the glucan products of WT mSBEIIa and the mutants R363K and R456K showed more than one peak, which agrees with the previous result on the WT mSBEIIa observed by Hernandez et al. [41]. This suggests that mSBEIIa can efficiently produce more than a single narrow range of chain lengths. The largest chain-lengths produced by R363K mSBEIIa, which showed a lower activity compared to WT mSBEIIa, were centered around DP140 (the largest peak observed from R363K mSBEIIa in Fig 3). However the length of the binding groove of the mSBEIIa homology model was approximately 46 Å [57]. Given that an individual glucose unit is about 6.3 Å in length, the binding groove itself is expected to only accommodate a maximum of 8 glucose units. In order to give rise to the preference to transfer DP 140 chains, it is possible that the glucan chain binds to other sites on the enzyme surface a long distance from the active site domain. In this case the branches could also come into contact and bind to the N-terminal domain of mSBEIIa. This is consistent with the observation from the crystal structure of rice SBEI in a complex with maltopentaose and glucose, which suggests there are three binding sites outside the catalytic domain located near the *N*-terminus, one of them being a carbohydrate-binding module (CBM): CBM48, http://www.cazy.org [35]. Indeed, the N-terminal domain of SBE has been shown to play a major role in determining the length of transferred chain [58, 59]. The binding sites in the N-terminal domain may have different functions and affinities towards different types of starch, as for example has been seen in the barley α-amylase [60]. These binding sites may also not be occupied simultaneously. Whether they are involved in binding a specific substrate would probably depend on the chain length and also kinetic factors such as the location on the substrate to which the enzyme initially binds. The involvement of more binding sites may facilitate the transfer of longer chains, especially where a long-chain substrate is involved. The number of binding sites involved would probably affect how strongly SBE binds to the substrate. Another possibility is that the enzyme slides along segments of the chain, cutting and joining the chain to form a branch leading to branches of differing length. In this scenario, the strength with which the SBE binds to the substrate would also be expected to alter the branch length. In either case, we would suggest that the different peaks in the SEC weight distribution depend on which of the N-terminal sites are involved in binding.

There is however a minimum on the number of binding subsites essential for binding oligosaccharides within the catalytic domain, which must be *X*_0_+*X*_min_ (Fig 1) [40]. From the FACE results, the minimum chain length that mSBEIIa can crop is DP 12, suggesting that the number of essential binding subsites for mSBEIIa is 12 (= *X*_0_ + *X*_min_).

The observation that SBEIIa has a preference for transferring intermediate chains explains the findings with the plant mutants having a lesion in SBEI. In both monocots and dicots, either down-regulation or elimination of SBEI activity alone has minimal effects on starch synthesis and composition in tubers, leaves, and endosperm, respectively [31, 32]. It is evident that the loss of SBEI has virtually no functional impact. This suggests that the role SBEI plays in starch biosynthesis can be fully compensated by other branching enzymes, which implies in turn that either SBEIIa or SBEIIb can transfer longer chains when necessary. The N-terminal domain of SBEII may be involved in this, as suggested above. By contrast, loss of BEII is only partially complemented by other SBE isoforms. Further investigation of the differences in the catalytic domain and N-terminal domain between SBEI and SBEII may help explain these findings.

### Disruption of the catalytic site associated with R456K leads to variation in the DP during branch formation

**Fig 8:**
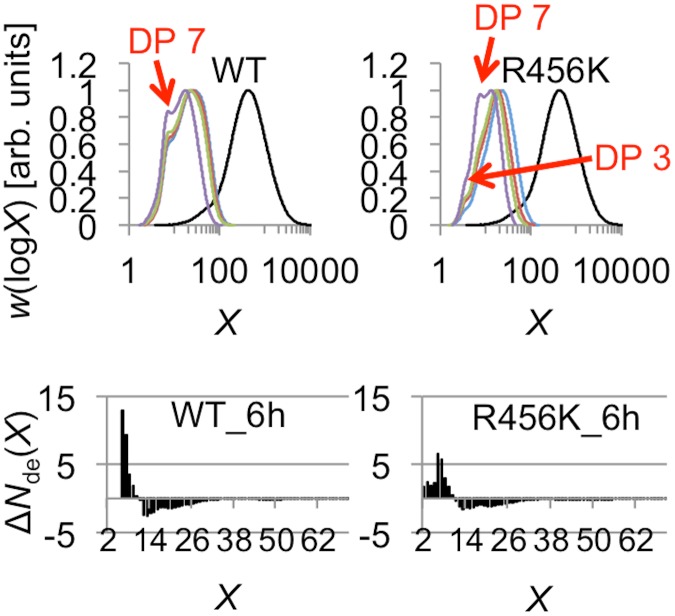
Comparison of chain profiles of the branched glucan products from WT and R456K mSBEIIa. The chain profiles were from SEC weight distributions of the glucan products of WT and R456K mSBEIIa from Fig 3 and the FACE number distributions of the glucan products after 6 h incubation with WT and R456K mSBEIIa from Fig 4. The different peaks are highlighted with their DPs in the SEC weight distributions. Black, blue, red, green, and purple lines in the SEC weight distribution are for CLDs after 0, 3, 6, 9 and 24 h incubation, respectively.

Characterization of the glucan products obtained with R456K mSBEIIa using both SEC and FACE revealed a DP distribution that differed significantly from that of WT mSBEIIa. This is most evident in the FACE data, which showed values in the range DP 2–5 (Fig 8). This suggests that R456K was able to create shorter chains than the wild type and that the length of the chains created corresponds to a broad range, as opposed to being primarily DP 6–7.

The MD simulations suggest that the equivalent mutation to R456K in rice SBEI, namely R342K, leads to major changes within the active site. Specifically, Glu399, which formed stable hydrogen bonds with the substrate in the wild-type, interacted instead with Lys342 in the mutant. In addition, Tyr235, which interacted with His467 in the wild type, also interacted with Lys342 in the mutant, affecting the interaction of His467 with the substrate. It is possible that these changes could increase the variability in the position at which the starch chain is cut and thus would explain the SEC and FACE data, in the case of R456K mSBEIIa. Although the mutation R456K leads to more variability in the position at which the chain is cut, the total number of binding subsites was apparently unchanged. The minimum chain length that R456K mSBEIIa could cut MAZACA amylopectin was the same as for WT mSBEIIa (DP12). Thus in the case of R456K mSBEIIa, *X*_0_ and *X*_min_ may vary (2<*X*_min_<10 and 2<*X*_0_<10), but the sum of *X*_0_ and *X*_min_ is still 12.

The site corresponding to Arg456 in mSBEIIa also seems to play an important role in orienting the catalytic site in other BEs for which crystal structures are available. For example, in *E*. *coli* GBE, the corresponding site is Arg403 (*E*. *coli* numbering). This residue is highly conserved and lies very close to Asp405 (*E*. *coli* numbering) one of the catalytic residues [61]. The corresponding residue in the *Mycobacterium tuberculosis* H37Rv GBE is Arg409 (*M*. *tuberculosis* numbering). This residue is also conserved and lies close to the nucleophile residue Asp411 (*M*. *tuberculosis* numbering) [62]. In fact the position and orientation of the corresponding Arg residues from SBE, isoamylase, α-amylase and CGTase suggests that this residue is conserved in all structures [63–65].

Considering that the residue of Arg456 (maize SBEIIa numbering) is conserved amongst different SBEs (refer to the sequence alignment in the SI), the effects of the mutation of Arg to Lys at this position could have similar effects on the transferred chain length of different SBEs. This is of particular interest because the different transferred chain length feature of SBEs will most likely develop a different amylopectin structure in plants, which is related to the nutritional properties of starch.

The enzyme assay on R456K mSBEIIa showed that it retained ~27.8% of the original activity of the WT mSBEIIa. The native affinity gels showed the mutation also affected the binding affinity of mSBEIIa for linear branches. These suggest that this residue is involved in maintaining both the catalytic function and substrate binding of mSBEIIa. However the mutations on the corresponding site of maize SBEIIb suggest that this site is important for the catalytic function of SBEIIb but may not be directly involved in substrate binding [66, 67].

### Mutation effects on the enzymatic activity

Mutation of Tyr352 and Glu513 led to the decrease in the activity of mSBEIIa but their effects on the transferred chain length of mSBEIIa could not be determined because of their insufficient activity (Fig 4). That the mutations affected the activity of the enzyme is not surprising, as the equivalent residues in other BEs are involved in catalysis and substrate binding [34, 61, 62]. In particular, Glu513 is responsible for the protonation and deprotonation necessary on the leaving group and attacking oxygen. Both sites are located in the catalytic center around subsites—1 and +1. This is supported by the native affinity gel, Fig 5, which suggests that mutations of Y352F and E513D lowers the binding affinity of mSBEIIa for a linear glucan. This is also supported by other mutation studies. The replacement of the equivalent residue to Tyr352 in *E*. *coli* GBE (Tyr300, *E*. *coli* GBE numbering) by Ala, Asp, Leu, Ser, or Trp, resulted in mutant enzymes with less than 1% of the original activity [68]. Although the conservative substitution by functionally similar amino acid Phe retained 25% of the original activity, the thermal stability of Y300F *E*.*coli* GBE was lowered significantly. The substitution of the residue corresponding to Glu513 in maize SBEIIb (Glu502, maize SBEIIb numbering based on the sequence alignment in SI) by either Gln or Asp resulted is a loss of enzymatic activity [69].

The mutation at Ser349 and Arg363 also changed the activity of mSBEIIa, with S349F resulting in the inactivation of mSBEIIa. Ser349 and Arg363 are not well studied in the literature. However, the homology model for mSBEIIa generated by SWISS-MODEL (Fig 2) suggests that Ser349 is located in the catalytic groove of mSBEIIa. Thus, it is possible that the replacement of Ser by the much larger Phe could easily inactivate mSBEIIa. However this mutation did not significantly change the binding affinity of mSBEIIa towards linear glucan, as shown in Fig 5. Arg363 is located on the back of the binding groove of mSBEIIa (Fig 2) and as expected had a relatively small direct effect on the activity of mSBEIIa. R363K has 55.5% of the enzymatic activity of WT mSBEIIa. This result is consistent with studies on maize SBEIIb [66]. When Arg363 was replaced by Ala in maize SBEIIb, the activity of the mutated enzyme was comparable to that of WT maize SBEIIb. However the native affinity gel showed that R363K changed the affinity of mSBEIIa for the linear glucan. This is a strong indication that Arg363 is involved in the substrate binding, which further suggests that longer starch chains bind by wrapping around mSBEIIa, not just in the binding groove. The transferred chain length of R363K mSBEIIa was the same as WT mSBEIIa (Fig 4). The effect of S349F mSBEIIa on the transferred chain length could not be determined, as the activity was insufficient (Fig 4).

### Predicting amylopectin CLD from the mutated mSBEIIa

Although changing one enzyme without affecting other enzymes in plants is hard, especially when considering the possibility that starch biosynthetic enzymes work in complexes, it is helpful to have a simple prediction before undertaking the long process to develop transgenic plants. From the prediction, the mutants from this study have a good opportunity to produce a significantly different starch structures in plants by their changed activity and transferred chain length.

## Conclusions

The characterization of the *in vitro* branching products of mutated mSBEIIa by SEC and FACE suggests amino acid residues that are important for the activity and transferred chain lengths of mSBEIIa. Considering that these residues are highly conserved amongst different BEs, they are most likely to have the same effects on other BEs. This could therefore offer a general approach for the engineering of SBEs in order to optimize the starch structure in plants, for example to obtain starch with longer chains. Of course, the effects of the mutations examined here must still be tested in plants. Nevertheless, the results presented here confirmed the prediction from the computational model used in this work that adjusting the different parameters of SBE(i) (i.e. β_(i)_ and *X*_min(i)_) could result in quantitatively different CLD and that the mutation of SBE can result in different starch structures. Overall, the combination of methods described provides a novel basis for the development of an improved understanding of starch biosynthetic enzymes.

## Supporting Information

S1 Fig Sequence alignment of mSBEIIa among other 14 different branching enzymes by Clustal Omega. Ecoli_GBE (*Escherichia coli* GBE), H37RV_GBE (*Mycobacterium tuberculosis* H37RV GBE), Rice_SBEI (Rice (*Oryza sativa Japonica Group*) SBEI), Wheat_SBEI (Wheat (*Triticum aestivum*) SBEI), Wheat_SBEIIa (Wheat (*Triticum aestivum*) SBEIIa), Wheat_SBEIIb (Wheat (*Triticum aestivum*) SBEIIb), Barley_SBEIIa (Barley (*Hordeum vulgare subsp*. *vulgare*) SBEIIa), Barley_SBEIIb (Barley (*Hordeum vulgare subsp*. *vulgare*) SBEIIb), Maize_SBEI (Maize (*Zea mays*) SBEI), Maize_SBEIIb (Maize (*Zea mays*) SBEIIb), Pea_SBEI (Pea (*Pisum sativum*) SBEI), Pea_SBEII (Pea (*Pisum sativum*) SBEII), Potato_SBEII (Potato (*Solanum tuberosum*) SBEII), and Arabidopsis_SBEII (Arabidopsis (*Arabidopsis thaliana*) SBEII) were used for the sequence alignment. An * (asterisk) indicates positions which have a single, fully conserved residue. A : (colon) indicates conservation between groups of strongly similar properties—scoring >0.5 in the Gonnet PAM 250 matrix. A. (period) indicates conservation between groups of weakly similar properties—scoring ≤0.5 in the Gonnet PAM 250 matrix. The five mutated sites of mSBEIIa are highlighted in the red box.
(DOCX)

S2 Fig SDS-PAGE of purified ~100kDa mSBEIIa enzymes. Lanes 1 to 7 are protein ladder, WT, Y352F, E513D, S349F, R363K, and R456K mSBEIIa, respectively. The sizes of the standard proteins in lane 1 are labeled on the left.
(DOCX)

S1 Text Molecular dynamics simulations. (DOCX)
