# Supplementary material for: The Characterization of Modified Starch Branching Enzymes: Toward the Control of Starch Chain-Length Distributions
Source: PLoS One. 2015 Apr 13;10(4):e0125507. doi: 10.1371/journal.pone.0125507 (PMC4395411; doi:10.1371/journal.pone.0125507)
Supplement: S1 Fig — Ecoli_GBE (Escherichia coli GBE), H37RV_GBE (Mycobacterium tuberculosis H37RV GBE), Rice_SBEI (Rice (Oryza sativa Japonica Group) SBEI), Wheat_SBEI (Wheat (Triticum aestivum) SBEI), Wheat_SBEIIa (Wheat (Triticum aestivum) SBEIIa), Wheat_SBEIIb (Wheat (Triticum aestivum) SBEIIb), Barley_SBEIIa (Barley (Hordeum vulgare subsp. vulgare) SBEIIa), Barley_SBEIIb (Barley (Hordeum vulgare subsp. vulgare) SBEIIb), Maize_SBEI (Maize (Zea mays) SBEI), Maize_SBEIIb (Maize (Zea mays) SBEIIb), Pea_SBEI (Pea (Pisum sativum) SBEI), Pea_SBEII (Pea (Pisum sativum) SBEII), Potato_SBEII (Potato (Solanum tuberosum) SBEII), and Arabidopsis_SBEII (Arabidopsis (Arabidopsis thaliana) SBEII) were used for the sequence alignment. An * (asterisk) indicates positions which have a single, fully conserved residue. A : (colon) indicates conservation between groups of strongly similar properties—scoring >0.5 in the Gonnet PAM 250 matrix. A. (period) indicates conservation between groups of weakly similar properties—scoring ≤0.5 in the Gonnet PAM 250 matrix. The five mutated sites of mSBEIIa are highlighted in the red box. (DOCX) [file pone.0125507.s001.docx]

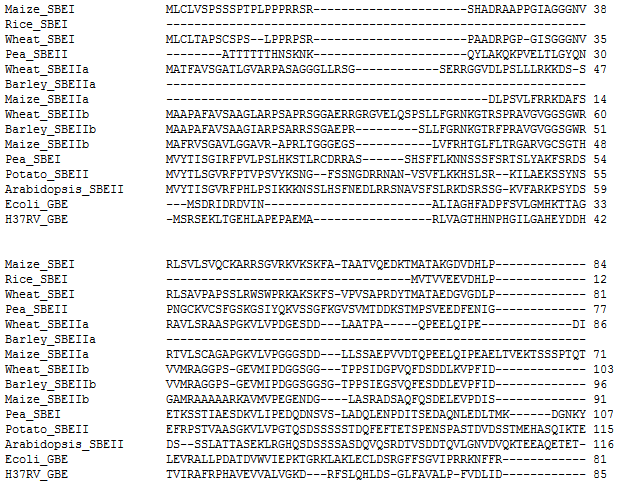


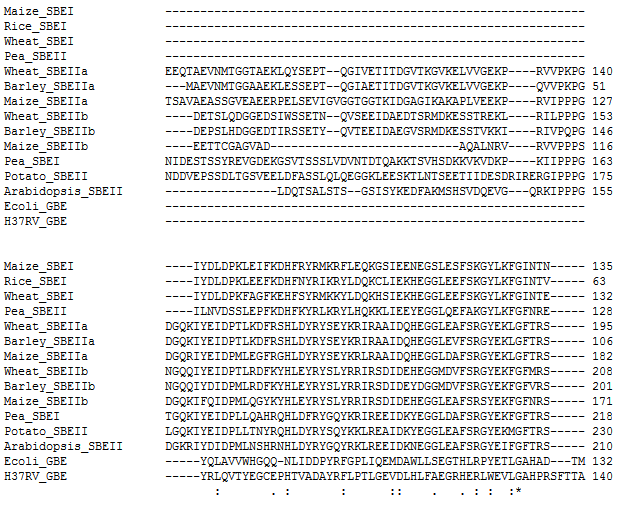


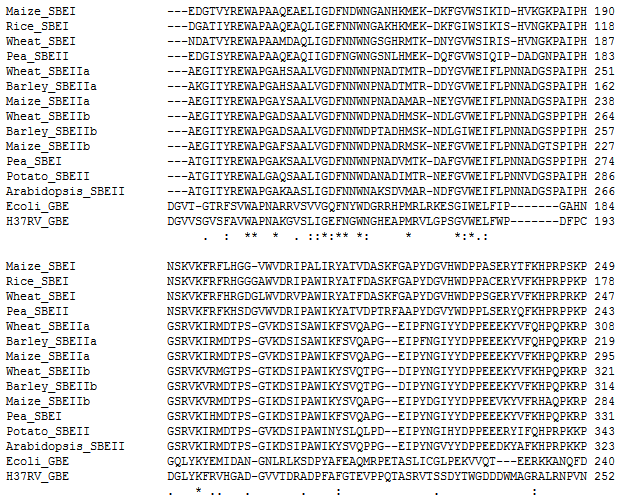


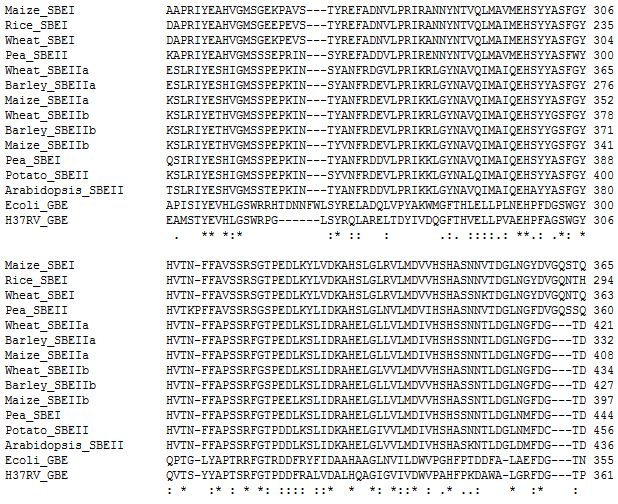


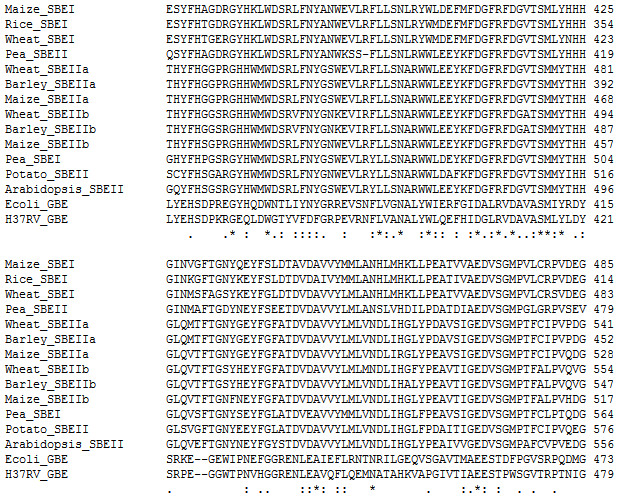


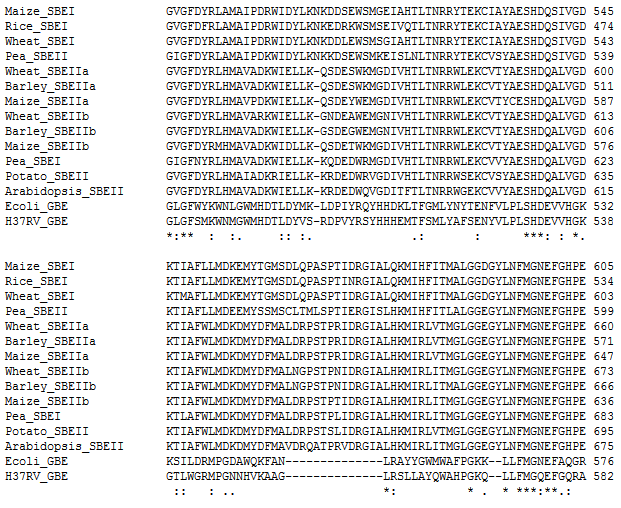


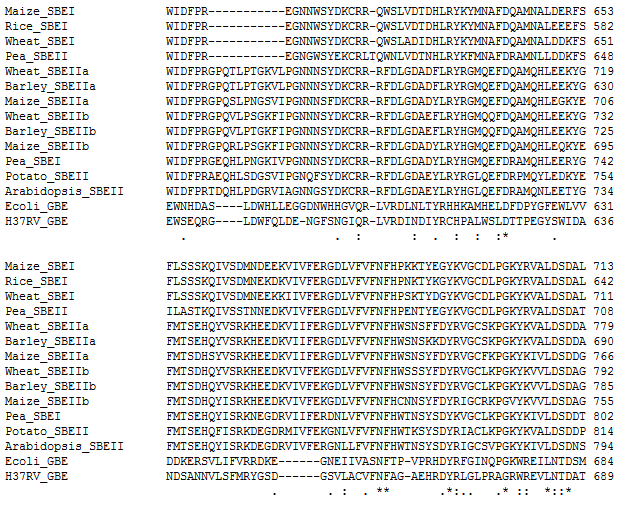


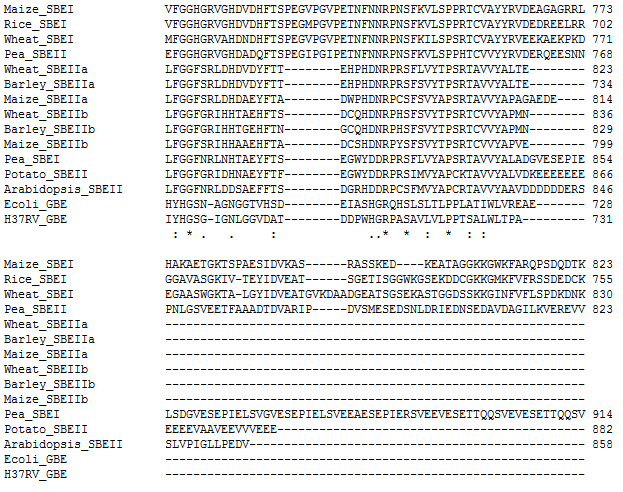


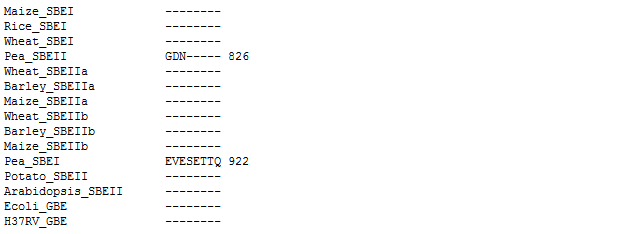


**Figure S1: Sequence alignment of mSBEIIa among other 14 different branching enzymes by Clustal Omega.**
