# Supplementary figures and images for: The Characterization of Modified Starch Branching Enzymes: Toward the Control of Starch Chain-Length Distributions
Source: PLoS One. 2015 Apr 13;10(4):e0125507. doi: 10.1371/journal.pone.0125507 (PMC4395411; doi:10.1371/journal.pone.0125507)

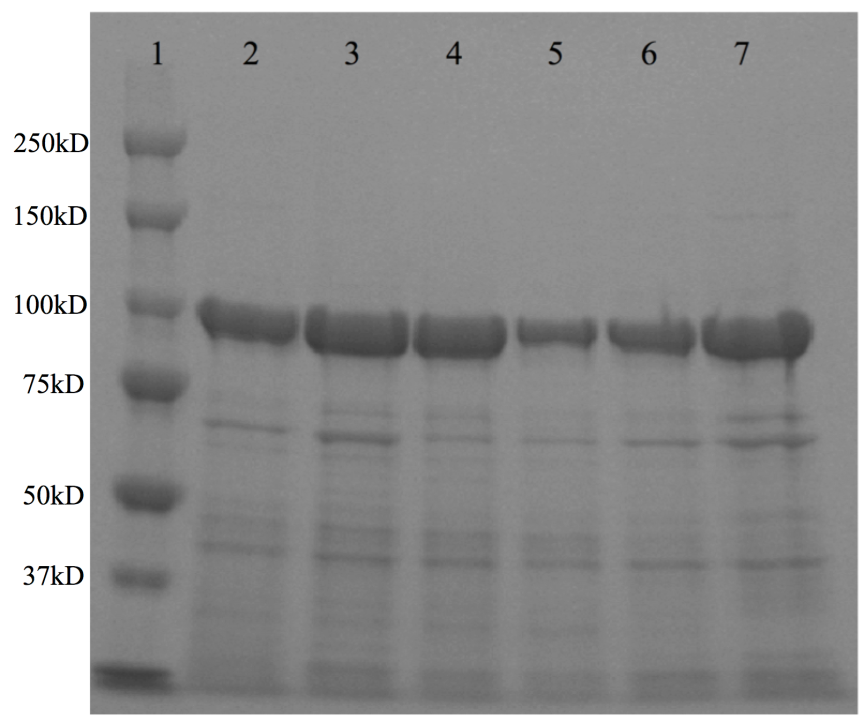


**Figure S2. SDS-PAGE of purified ~100kDa mSBEIIa enzymes.**

Supplement: S2 Fig — Lanes 1 to 7 are protein ladder, WT, Y352F, E513D, S349F, R363K, and R456K mSBEIIa, respectively. The sizes of the standard proteins in lane 1 are labeled on the left. (DOCX) [file pone.0125507.s002.docx]
