# Supplementary material for: The Characterization of Modified Starch Branching Enzymes: Toward the Control of Starch Chain-Length Distributions
Source: PLoS One. 2015 Apr 13;10(4):e0125507. doi: 10.1371/journal.pone.0125507 (PMC4395411; doi:10.1371/journal.pone.0125507)
Supplement: S1 Text — (DOCX) [file pone.0125507.s003.docx]

## Molecular dynamics simulations

All MD simulations were performed using the GROMACS simulation program version 3.3.3 [[1](#_ENREF_1)], with the GROMOS54A7 force field for protein and starch molecules [[2](#_ENREF_2)]. Two systems were simulated. The first consisted of a complex of wild-type rice SBEI with maltopentaose, and the second a complex of R342K rice SBEI with maltopentaose. The starting structures for both systems were based on the crystal structure of SBEI from rice (*Oryza sativa L.*) SBEI (PDB ID 3AML). This structure does not contain substrate although some density of unknown origin in the proposed binding groove was observed. For this reason, maltopentaose was docked into the binding groove using AutoDock Tools 4 (ADT4) centered on the region of the observed density. In both cases the C-terminal domain consisting of residues 583–698 was truncated in order to decrease the size of the system and the associated computational time. This truncation was found to have no effect on the structure of the central catalytic domain (results not shown). The truncated native and mutated proteins, including the docked sugar, were placed at the center of a dodecahedron box in which the distance between the parallel faces was 10 nm and solvated with ~21000 simple point-charge (SPC) water molecules [[3](#_ENREF_3)]. The protonation states of titratable residues were set appropriate to pH 7.5. Histidine residues were singly protonated (neutral) with the location of the proton assigned manually depending on the local environment. The total charge of the system was neutralized by adding 10 Cl– counter ions. The Berendsen weak coupling method was employed to maintain the temperature and the pressure of the system at 300 K and 1 atm, with coupling times of 0.1 and 1.0 ps respectively [[4](#_ENREF_4)]. The SHAKE algorithm was applied to constrain the bond lengths within the system [[5](#_ENREF_5)]. The equations of motion were integrated using a time step of 2 fs. Non-bonded interactions were evaluated using the twin–range cutoff scheme. Short-range interactions within 0.9 nm were calculated every step and interactions formed between 0.9 and 1.4 nm were calculated every 5 steps. A reaction-field correction with an epsilon value of 78 was applied to correct for the truncation of electrostatic interactions beyond 1.4 nm. To initiate the simulations, the systems were first energy-minimized. The system was then equilibrated briefly with position restraints of 1000 kJ mol–1 nm–2 on all heavy atoms. The restraints were removed and each system simulated independently twice for 25 ns. The coordinates and energies were saved every 10000 steps for analysis. Topologies and parameters used for maltopentaose are available from the Automated Topology Builder (ATB) [[6](#_ENREF_6), [7](#_ENREF_7)].

1. Van Der Spoel D, Lindahl E, Hess B, Groenhof G, Mark AE and Berendsen HJC. GROMACS: Fast, flexible, and free. J Comput Chem. 2005;26(16): 1701-1718.

2. Schmid N, Eichenberger A, Choutko A, Riniker S, Winger M, Mark A, et al. Definition and testing of the GROMOS force-field versions 54A7 and 54B7. Eur Biophys J. 2011 2011/07/01;40(7): 843-856.

3. Berendsen HJC, Postma JPM, van Gunsteren WF and Hermans J. Interaction Models for Water in Relation to Protein Hydration. In: B. Pullman, editors. 21. Springer Netherlands; 1981. p. 331-342

4. Berendsen HJC, Postma JPM, Vangunsteren WF, Dinola A and Haak JR. MOLECULAR-DYNAMICS WITH COUPLING TO AN EXTERNAL BATH. Journal of Chemical Physics. 1984;81(8): 3684-3690.

5. Ryckaert J-P, Ciccotti G and Berendsen HJC. Numerical integration of the cartesian equations of motion of a system with constraints: molecular dynamics of n-alkanes. Journal of Computational Physics. 1977 3//;23(3): 327-341.

6. Lins RD and Hünenberger PH. A new GROMOS force field for hexopyranose-based carbohydrates. Journal of Computational Chemistry. 2005;26(13): 1400-1412.

7. Malde AK, Zuo L, Breeze M, Stroet M, Poger D, Nair PC, et al. An Automated Force Field Topology Builder (ATB) and Repository: Version 1.0. Journal of Chemical Theory and Computation. 2011 2011/12/13;7(12): 4026-4037.
